# The Role of Mitochondrial Dysfunction in Alzheimer’s: Molecular Defects and Mitophagy-Enhancing Approaches

**DOI:** 10.3390/life13040970

**Published:** 2023-04-08

**Authors:** Reem M. Farsi

**Affiliations:** Department of Biological Sciences, Faculty of Science, King Abdulaziz University, Jeddah 21462, Saudi Arabia; rfarsi@kau.edu.sa

**Keywords:** Alzheimer’s disease, mitochondrial dysfunction, mitophagy, therapeutic and nanotherapeutic approaches

## Abstract

Alzheimer’s disease (AD), a progressive and chronic neurodegenerative syndrome, is categorized by cognitive and memory damage caused by the aggregations of abnormal proteins, specifically including Tau proteins and β-amyloid in brain tissue. Moreover, mitochondrial dysfunctions are the principal causes of AD, which is associated with mitophagy impairment. Investigations exploring pharmacological therapies alongside AD have explicitly concentrated on molecules accomplished in preventing/abolishing the gatherings of the abovementioned proteins and mitochondria damages. Mitophagy is the removal of dead mitochondria by the autophagy process. Damages in mitophagy, the manner of diversified mitochondrial degeneracy by autophagy resulting in an ongoing aggregation of malfunctioning mitochondria, were also suggested to support AD. Recently, plentiful reports have suggested a link between defective mitophagy and AD. This treaty highlights updated outlines of modern innovations and developments on mitophagy machinery dysfunctions in AD brains. Moreover, therapeutic and nanotherapeutic strategies targeting mitochondrial dysfunction are also presented in this review. Based on the significant role of diminished mitophagy in AD, we suggest that the application of different therapeutic approaches aimed at stimulating mitophagy in AD would be beneficial for targeting or reducing the mitochondrial dysfunction induced by AD.

## 1. Introduction

Alzheimer’s disease (AD) is a prevalent and irretrievable neurodegenerative syndrome related to old age. It is described by two focal histological features: the gathering of Aβ (beta-amyloid), which accumulates in the hyperphosphorylation of TauP (Tau protein), and extracellular absentminded plaques, which create intracellular NFTs (neurofibrillary jumbles) [1,2,3]. The main clinical symptoms of this syndrome are a weakening of memory and cognitive abilities, accredited by intermittent AD to genetic and/or environmental vulnerability aspects, the first sign being the *APOE* (*Apolipoprotein* gene) [4]. Selectively, AD can target and destroy neuronal cells in the hippocampus, the zone of the neuron connected with memory, which is particularly susceptible [1]. Some mutations in familial AD, such as the *PSEN 1* and *2* (Presenilin 1 and 2) genes and APP (*Amyloid Precursor Protein*), may be linked with the expansion of AD.

**Figure 1:**
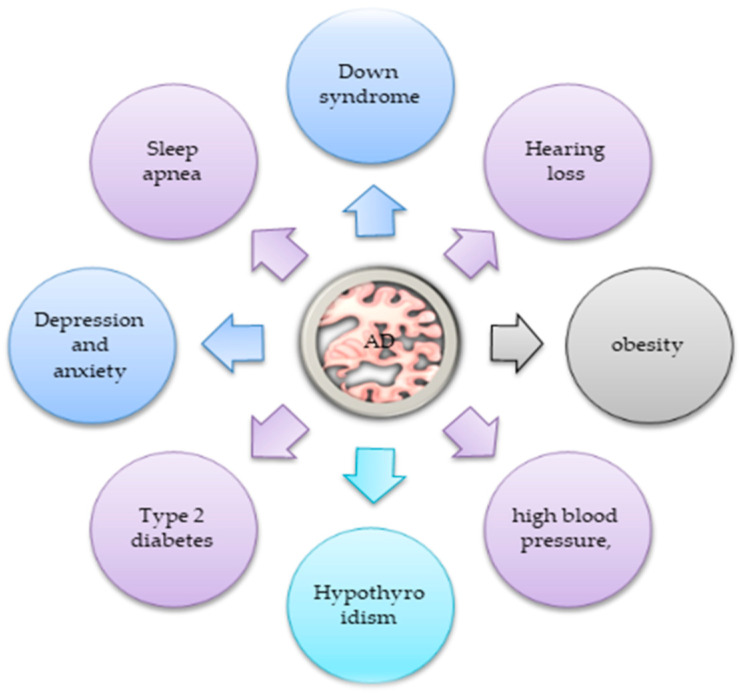
A summary of some of the symptoms accompanying AD, including sleep apnea, augmented anxiety and depression, obesity, memory loss, hypertension, type 2 diabetes, hypothyroidism, high blood pressure, Down syndrome, and hearing impairment.

Furthermore, recognizing AD-associated mutations can support the construction of the amyloidogenic pathway theory describing greater Aβ synthesis and its diminished authorization as the main activators of AD expansion [5]. Moreover, AD is a complex neurodegenerative syndrome, and the symptoms and features of AD are still not fully understood. Some of them are exhibited at the early stage of AD, such as mood and personality alterations, augmented anxiety, misplacing, poor judgment, and memory loss [6,7]. In the advanced stage, other signs appear, including removal from social events, amnesia, aberration and misuse, fluent aphasia, and psychiatric symptoms [7]. As one investigation progressed (Figure 1), it showed that more than a dozen syndromes associated with AD, comprising hypertension, obesity, type 2 diabetes [8], hypothyroidism, vitamin deficiency [9], Down syndrome, and hearing impairment [10], among others, reveal the menace of AD. For many decades, scientists have used several therapeutic strategies for treating or reducing the severity of AD, using some mitophagy enhancers to promote the mitochondria function in AD syndrome.

Mitochondria are self-division cellular components existing in cellular systems, excluding red blood cells. Mitochondria has numerous functions via channels and transporters positioned on their OM (outer membrane), ensuring the interactions of lipids, metabolites, and calcium (Ca^2+^) with the remnants within the cytosol and other cellular components.

Apoptosis-dependent mitochondrial events, and their exact dynamic fusion, mobility, degradation, and fission, are sensed at this OM. Most cellular bouncing loads are supplied by mitochondria, which produce ATP (adenosine triphosphate) via the stimulation of OXPHOS (oxidative phosphorylation). OXPHOS stirs in the inner membrane (IM) via two mobile electron carriers and five enzyme complexes. Beyond being the metabolite and energy wage earners, mitochondria produce oxidative stress [11], presenting as pathophysiological messengers of an excess of cell actions, including proliferation, apoptosis, immunity, and recognition [2]. As mentioned previously, the accumulation of Aβ and P-tau show large functions in the cellular alterations associated with AD, comprising neuronal loss, mitochondrial dysfunction, synaptic impairment, and malfunctioning mitophagy [12,13]. This process (mitophagy) is the cellular route by which scratched mitochondria are selectively detached, and it shows an imperative role in mitochondrial functionality [14].

Mitochondrial homeostasis is a state of equilibrium among the biogenesis of mitochondrial aspects and dysfunctions, such as autophagic degeneration [2], in addition to superfluous ones, via a discerning procedure mentioned concerning mitophagy paths [15]. Throughout this clearance cascade, insalubrious mitochondria are submerged via a phagophore that establishes the mitophagosome process, which will, at that juncture, consolidate with a lysosome to create a mitophagolysosome [2,16]. Mitochondrial damage manifests earlier in the pathological phase related to AD [17], and, therefore, may represent a “risk factor” of AD.

Results in cellular (in vitro) and animal (in vivo) models of AD, simulating familial types of AD patients, have formerly supported the assumption that damaged mitophagy. This feature is a significant characteristic of the syndrome. It could invent both the downstream and upstream movement of Tau and Aβ in a malicious sphere, eventually instigating cognitive shortfalls and synaptic dysfunctions [3]. This study concerns investigations that explore mitochondrial mitophagy’s position in patients with AD in addition to cellular (in vitro) and animal (in vivo) models with AD disease. Moreover, we depict the various approaches (lifestyle, pharmacological, and genetic) intended to support the elimination of excess mitochondrial dysfunction markers by mitophagy and focus on the positive properties they have on AD patients.

## 2. Research Strategy

A comprehensive data valuation was accomplished to consider all offered peer-reviewed papers that examined the mitochondrial function in AD and the mitophagy mechanisms. The criticism was revealed via endorsed records collected from databases, viz., MEDLINE, Google Scholar, Science Direct, Scopus, and Web of Science. Primary exploration keywords were defined as Alzheimer’s disease, mitochondrial dysfunction, mitophagy, and therapeutic and nanotherapeutic approaches.

## 3. Symptoms Features of AD

As is well described, AD is a complex neurodegenerative syndrome and the symptoms and features of AD are still not fully understood. Some of them are exhibited at the early stage of AD, such as mood and personality alterations, augmented anxiety, misplacing, poor judgment, and memory loss [6,7]. In the advanced stage, other signs appear, including removal from social events, amnesia, aberration and misuse, fluent aphasia, and psychiatric symptoms [7]. As an investigation progressed (Figure 1), it showed that more than a dozen syndromes, comprising hypertension, obesity, type 2 diabetes [8], hypothyroidism, vitamin deficiency [9], Down syndrome, and hearing impairment [10], among others, reveal the menace of AD.

## 4. Mitochondrion Impairments in AD

Anomalous mitochondrial assembly, the aggregation of injured mitochondria, weakened metabolic changes and bioenergetics, and exaggerated ROS synthesis are familiar in different AD examples [18]; therefore, they support possible molecular boards for the remedy of some disorders, such as AD. The conservation of purposeful mitochondrial occupants throughout the lifecycle of nerve cells is of dominant significance. It implicates an acceptable arrangement of mitochondrial cascades maintained by the synchronized interaction between the machinery of biogenesis and mitophagy [19] and mitochondrial splitting and synthesis [20].

**Figure 2:**
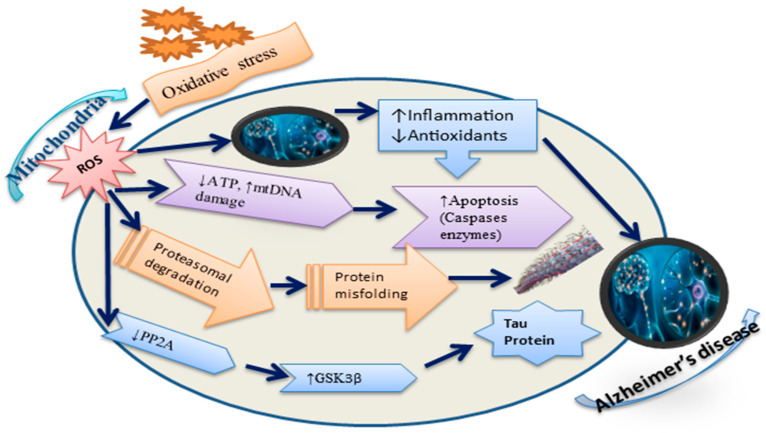
Mitochondria impaired in Alzheimer’s disease via ROS. First, the overproduction of ROS can decrease antioxidative defense and trigger inflammation. Moreover, the ROS can imbalance ATP production and decrease the mitochondrial ΔΨm by negatively affecting mitochondrial supplies, negotiated dynamics and apoptosis (increase caspase enzymes), and mitophagy manners, and disrupting ATP sources. Excessive ROS results in the reticence of PP2A (phosphatase 2A), which also stimulates GSK3β (glycogen synthase kinase), instigating tau protein synthesis and neurofibrillary tangles aggregation.

Consequently, it is facile to visualize in what way the intermission of any of these mitochondrial routes requires a robust effect on cellular attitudes and viability. Meanwhile, nerve cells, specifically due to inconsistency, need an exceptionally elevated level of energy support for the neurotransmission process, and they requirement a competent scheme to disregard injured mitochondria [21,22] and decrease ROS-instigated cell death [23]. Various reports have supported the identification of mitochondrial dysfunctions disturbing the quality regulator machinery of mitochondria [24], giving the vibrant suggestion that all complicated phases are compromised in AD pathogenesis [16,24]. Figure 2 depicts the causes of mitochondrial dysfunction pathways in AD.

### 4.1. Oxidative Stress and Mitochondrial Defects in AD

Reactive oxygen species (ROS) are demarcated as biologically oxidative markers (OS). The OS is instigated by an inequity between the synthesis and aggregation of ROS, which are unavoidable metabolism byproducts that characterize an ambiguous weapon in the cellular schemes under accurately controlled situations [25]. ROS can assist indispensable functions as, for instance, signing mediators, but can also impair the cellular components when formed at extreme levels; subsequently, they react with all main biocomponents, including lipids, nucleic acids (RNA and DNA), and proteins [12]. Either greater ROS or a weakened antioxidant scheme can slope the hemostasis of the cellular scheme concerning an oxidative complaint [26].

The cerebrum is particularly vulnerable to OS injury, owing to its significant amount of oxygen intake, eminent altitudes of PUFAs, and tremendous amounts of hemostasis-alterating metallic ions Furthermore, the neurons have a very inefficient defensive system [12,13]. As is well accepted, ROS has been confirmed to be responsible for cellular damage in older people and nerve cell complaints [27]. Indeed, the aggregations of Aβ protein instigated by ROS in AD produce lysosome sheath degeneration and ultimately participate in cellular apoptosis in brain tissues [26].

Moreover, an absence of CCO (cytochrome c oxidase), which is the greatest combined imperfection in the ETC (electron transport chain) in mitochondria within AD patients, results in an escalation in ROS synthesis, a reduction in energy supplies, and a disturbance of energy for metabolism in the cellular system, instigating cell death [28,29]. Additionally, ROS trigger the suppression of PP2A enzymes (phosphatase 2A) [27], which simplifies GSK-3β (glycogen synthase kinase 3β) activation and is related to tau phosphorylation. Subsequently, amplified GSK3β stimulation might generate neurofibrillary lesions and tau hyperphosphorylation in AD [13].

In the data, oxidative difference and a considerable escalation in its secondary products were frequently stated in AD. In addition, a great bulk of exploration has verified that lipid peroxidation (LPO), the development in which ROS use fats to yield LPO products through OS or ROS shackle response machinery, is critically greater in AD [26]. The most extensive LPO outputs considered in AD are responsive aldehydes, including acrolein (2-propenal), 4-hydroxynonal, and MDA (malondialdehyde), and metabolically and structurally constant iso-prostanoids, including F4-neuroprostanes and F2-isoprostanes. A considerable rise in MDA levels was described in the pyriform cortex, hippocampus [30], and erythrocytes in patients with AD.

Assessing MDA amounts, which are both cheap and easy to gather, might be of abundant significance for observing the development of AD and, thus, avoiding or treating it [31,32]. Contrarily, the OS indices that are regularly applied in biological specimens comprise TBARS (thiobarbituric acid-reactive constituents), free fatty acid statements, 2-propen-1-al (acrolein), neuro and iso-prostane synthesis, and HNE (4-hydroxy-2-*trans*-nonenal) for LPO; 3-NT (3-nitrotyrosine) and protein carbonyls (PC) for protein oxidation; progressive glycation completion molecules for carbohydrates; and 8-hydroxydeoxyguanosine (8-OHdG), 8-hydroxy-2’-deoxyguanosine (8-OHdG), other oxidized sources, and changed DNA restoration machinery for RNA and DNA oxidation [31]. Recently, the augmentations levels of toxic carbonyls, such as HNE and 3-NT, are among the earliest modifications realized after instigating the OS affront in AD [33].

In addition to previous categories, ROS contains both radical and non-radical ROS classes formed by an incomplete decrease in oxygen, such as hydroxyl radical (HO), superoxide hydrogen peroxide (H_2_O_2_), radical anion (O_2_), peroxynitrite (ONOO-), and nitric oxide (NO). As mentioned by other authors [31,34], the main site for the generation of OS is mitochondria through OXPHOS, in which electrons escape from the ETC in the mitochondrial cavity for generating the construction of O_2_. Moreover, protein nitrosylation/oxidation can also produce in methionine oxidation and *S*-nitrosylation (sulfoxidation), and the latter is the creation of the response between N^2^O^3^ and cysteine moiety to produce an SNO *(S*-nitrosothiol) [25,32]. The SNO is significant in regulation-instituted intracellular pathways, and the transformed SNO state was acknowledged in AD [33]. In patients with AD or the elderly, advanced mitochondrial dysfunction was associated by way of the principal origin of ROS formation; mitochondria are the main marker of oxidative impairment in the cellular scheme [22]. In light of this previously mentioned sensation, frequent reports have recognized mitochondrial dysfunction via the irregular treatment of ROS as an indispensable issue in the pathogenesis of AD [25,26,30].

Similarly, adding Aβ as oligomers with a bilayer can produce ROS expansion, thus introducing LPO in cellular membranes monitored by intracellular nucleic acid and protein oxidation [32]. It is imperative to footnote that OS is interrelated with mitochondrial aspects because mitochondria produce OS. Nevertheless, OS can source the weakening of mitochondrial assembly (Figure 2). To achieve the difficult balance in this system and diminish ROS points, modifying approaches, such as lifestyle and antioxidant medications, may support the protection of neuronal mitochondria from OS or ROS and, therefore, decrease the severity of AD.

### 4.2. Malfunctioning Energy Metabolism of Mitochondrial in AD

As stated in another section of this review, AD patients display primary metabolic discrepancies connected with mitochondrial dysfunction and the abnormal aggregation of injured mitochondria [35]. These features can be employed before clinical anomalies or histopathological examinations [35]. The shortcomings in the metabolism of energy sources in mitochondria are associated with AD occurrence [36], leading to neuron death. Unfailingly, the events of MRC enzymes (mitochondrial respiratory complexes) [37] and other mitochondria enhancers or nutrients, such as α-ketoglutarate dehydrogenase, pyruvate dehydrogenase compound, isocitrate dehydrogenase, and ATP synthase levels, were evidenced to be decreased. It was supposed that these formal activities are related to the mitochondrial damage of OXPHOS and Δψm, decreased ATP amounts, and augmented OS [37], which strengthens the statement of Aβ, provoking cognitive impairments in AD rats and participation in the polymerization and phosphorylation of Tau protein [38].

Similarly, reports show glucose hypometabolism, which is instigated by neuronal loss and decreased glycolysis. Synaptic dysfunction is noticeably apparent in the cortical and hippocampus districts [39], and precedes the clinical identification of AD by antedating the beginning of histopathological indications and structures [35,40]. It also leads to cognitive weakening in regular aging and advances mental decay from slight cognitive loss to the AD phase [40].

With this detrimental effect in mind, [41] explored how the metabolism of glucose differs in several stages of AD syndrome. The authors detected that, in the primary stage of cell death, a reduction in the progression of AD was found in the delayed stage, and glucose absorption was heightened, i.e., important proteins metabolizing and internalizing glucose, including phosphofructokinase, hexokinase, and glucose transporter, are up-regulated, in accompaniment with an escalation in L-lactate aggregation and a comparable reduction in oxygen uptake by mitochondria. Significantly, these neurons, demonstrating an alteration in the metabolism of glucose to approve lactate formation, are selected for confrontation alongside Aβ poisoning [42], and, as a consequence, mitochondria-generated ROS, faithfully connected with the toxicity of Aβ, lessened in resistant-cells with deference to delicate ones [43,44]. The discovery of how neurons show resilience to Aβ noxiousness will be crucial to illustrating how certain neurons within the patient’s brain with AD are capable of surviving when the majority die [18,30,45]. While the biological significance of the current verdicts based on an in vivo AD model is unidentified, the description of the machinery through which glycolysis paths triggered the upregulated cellular resistance to Aβ toxicity could offer potential goals for drug remedy in the management of AD at the primary phases [14].

In contrast, at the dawning stage of apoptosis [41,46], the damage of the adaptive benefit given via aerobic glycolysis aggravates the pathological procedures of fundamental neuron syndromes, predictably resulting in the death of the cell. Reports have revealed that the failure of glucose metabolism in the brain tissues occurs in the prodromal phases of AD and is more embroidered after the syndrome begins [47]. These reports [25,32,47] evidenced inadequate mitochondrial energy formation and cerebral glucose hypometabolism, and participation in synapse damage, while also suggesting the presence of the Tau and Aβ42 proteins in the brain’s neurons. Contrarily, the aggregation of Tau and Aβ42 generates mitochondrial impairment, modifies energy construction, and raises OS [45]. In addition, neurotoxic proteins also constrain glucose GLUT4 (transporter type 4) [48,49] and phosphofructokinase, delaying glucose intake, ATP synthesis, and aerobic glycolysis. These facts clearly indicate that the collaboration of these proteins is a source of neurodegenerative syndromes, supporting the assumption that mitochondrial impairment is an early instigator for AD [48], and creating an imperative to progress influential medications that relieve the bioenergetic shortfalls in susceptible nerve cells from the utmost AD-affected brain districts.

### 4.3. Mitophagy Dysfunction in Patients AD

**Figure 3:**
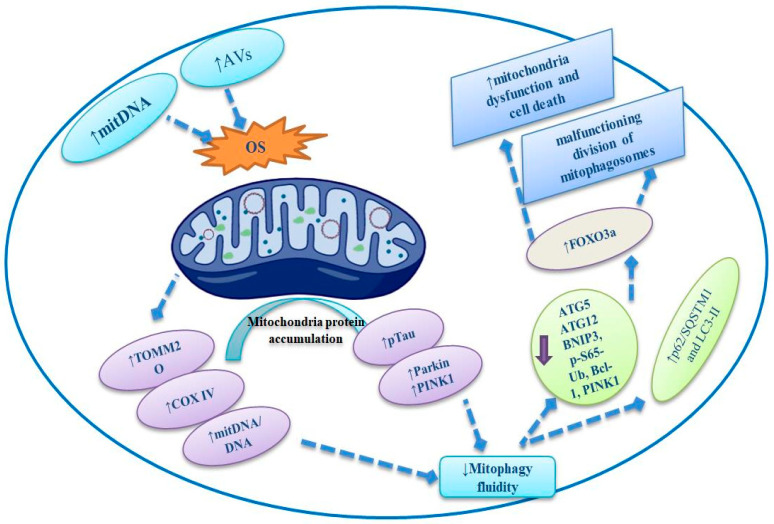
Aggregation of proteins and mitDNA (mitochondria DNA copy number) in the cytoplasm and in AVs (autophagic vacuoles) in the nerve cells of AD patients, introducing augmented oxidative injuries and mitochondrial proteins, such as TOMM20, COX IV, Parkin/PINK1, and pTau, and finally causing mitophagy fluidity. Moreover, a decrease in the expression of BNIP3, p-S65-Ub, Bcl-1, PINK1, and ATG12 and an escalation of the planes of p62/SQSTM1 and LC3-II have been detected in neuron tissues of patients with AD-inducing FOXO3a, promoting the malfunction of mitophagy and causing mitochondrial death.

In the year 2000, [50] was the first study to detect the impairment of the mitophagic pathway in AD. The same researchers revealed that the aggregation of proteins and mitDNA (mitochondria DNA copy number) in the cytoplasm and in the AVs (autophagic vacuoles) of the nerve cells of AD patients introduced augmented oxidative injuries. Depending on the clinical reports, it has been exhibited that augmented amounts of mitochondrial proteins, such as TOMM20 and COX IV, and an eminent proportion of mitDNA/nuclear DNA in AD neurons unravel [51]. Other outcomes maintained decreased mitophagy retrogression in patients’ neurons with significant amounts of pTau protein, indicating the involvement of Tau protein in this phenotype, as evidenced by [3,51]. The high contents of Parkin and PINK1 at early and late AD phases, respectively, and an escalation of mitochondrial indicators at both late and early phases were described as being present in AD hippocampi [52]. This could suggest decreased mitophagic fluidity connected with a deficiency in the initiation of Parkin/PINK1 signaling (Figure 3). The same pathways were also evidenced by other authors [53,54]. Similarly, some studies have clarified the downregulation of some mitophagy and autophagy genes, including Optineurin (OPTN), BNIP3L, FUNDC1, VDAC1, Bcl-1 (Beclin-1), ULK1, PI3K class III, ATG5, ATG12, BNIP3, AMBRA1, and VCP/P97, which are associated with AD-affected brains [54]. Consequently, a decrease in the expression of BNIP3, p-S65-Ub, Bcl-1, PINK1, and ATG12 and an escalation of the planes of p62/SQSTM1 and LC3-II have been detected in neuron tissues of APOEε4 heterozygote patients with AD disease (Figure 3) [53]. This last feature was believed to be identified by promoting the sluggish form of the FOXO3a transcript element repossessed in the cytosol [55]. Additionally, this mitophagy decrease was categorized by the aggregation of functionally and structurally impaired mitochondria (disordered cristae, decreased size, and squat ATP synthesis) and by a diminishing of the beginning stages of the mitophagy progression (decreased triggered LC3 employment to mitochondria and diminishing AMPK (AMP-activated protein kinase) signaling, categorized by AMPK phosphorylation, as well as the suppression of its board TBK1 and ULK1 pathways [43]. Hence, these explanations described a malfunctioning division of mitophagosomes with lysosomes [43] and an aggregation of scratched mitochondria in AVs as presented in the neuron tissues of patients with AD [56]. Remarkably, the malfunctioning mitophagy in a regiment of human SAD neurons was sustained by a rise in p62, LC3-I, and LC3-II levels and a decrease in Parkin and PINK1 levels in mitochondria-interlaced segments [57]. There is great value in seeing that all mitophagy indicator differences related to APP-CTF aggregation, especially concerning pTau and Aβ in an amount only connected to amplified LC3-II/I proportions and decreased Parkin, respectively, indicate a greater relation of sequestration miscarriage in mitochondria with APP-CTF aggregation in the neurons of people with AD in the brain tissues [35]. Uniquely, they established an innovative function for DISC1 as a mitophagy receptor existing in both the IMM and OMM that made phagophores impair mitochondria functionality and assembly via attaching to LC3-II with its LC3 motif [57]. Moreover, the diminished action of mitochondrial multiplexes I-V happens as early as AD phases I-II [35] in the EC (entorhinal cortex), indicating that mitophagy failure and mitochondrial impairments follow principally in susceptible brain districts. These modifications can be augmented by Aβ and participate in pTau aggregation through the initial phases of AD occurrence [3,35,51].

### 4.4. AD Patient-Derived Fluids and Cells

As seen in several AD patients, skin fibroblasts show dysfunctional mitophagy and autophagy exemplified by the decreased creation of Avs, the inferior quantity of lysosomes (LYS), and the aggregations of TOMM20 mitochondrial indicators [52]. Furthermore, Parkin altitudes are decreased in the mitochondria-fortified portion in response to the mitophagy prompt attached to the uncoupler of mitochondrial sheaths, which are recognized to activate PINK1 maintenance to the mitochondria and, thus, augment phosphorylation and Parkin recruitment [52]. The authors of [54,58] confirmed that SAD fibroblasts create “aged mitochondria” and have a decreased capability to mortify them. This characteristic has been additionally maintained by applying a MitoTimer analysis that permits the monitoring of mitochondria development by reporting the modification of wavelength production. This established that fibroblasts from vigorous personalities exhibit a longitudinal development ascent with a centralization of early mitochondria at the border and elder mitochondria neighboring the nucleus.

Uniquely, the fibroblasts of SAD do not show, for example, apportionment, but do slightly display changed mitochondria transportation to the retrogression location and a gathering of declininf mitochondria indices [54,58]. Furthermore, the study validated the weakened mitophagy and autophagy in the fibroblasts of an A246E (AD patient harboring a PS1 mutation) variant and in iPSC (pluripotent stem cells)-isolated nerve cells [59]. In this regard, the equal replicas exhibited improved LC3-II amounts and lessened LYS degeneration abilities revealed by the aggregations of both TOMM20 and p62. Although in conjunction with different SAD fibroblasts, Parkin amounts were augmented in FAD PS1 (A246E) fibroblasts related to non-isogenic observations [59]. To date, these clarifications have to be additionally established utilizing isogenic standers. Investigations also describe lysosomal blemishes in the FAD fibroblasts of humans with other *PS1* mutations connected with decreased levels of cathepsin D, lysosomal alkalization, and generally decreased macroautophagic squalor [21,58].

**Figure 4:**
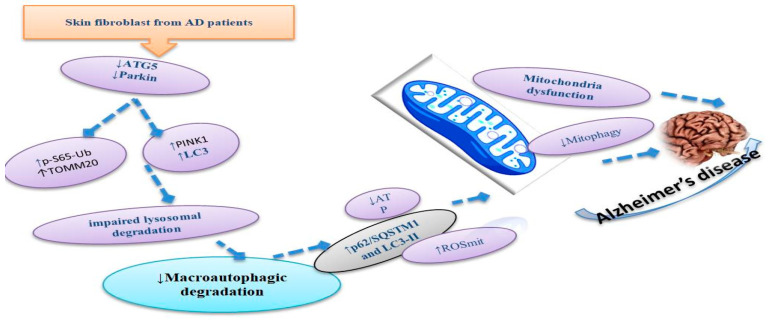
Mitophagy failure and mitochondrial dysfunction in iPSC-derived cortical neurons obtained from some cell mines and fluids of patients categorized by reduced levels of Parkin and ATG5, which induced an increase in PINK1, LC3, P-S65-Ub, and TOMMO20 in the mitochondria cavity, thus reducing the macroautophagy degradation. Moreover, all pathways might decrease the mitophagy process in the mitochondria of neurons in patients with AD.

Moreover, iNSC (iPSC-isolated nerve) blocked (KI) for PS1 (M146L) also revealed a decline of autophagy indices categorized via ULK1 stimulation and reduced LC3 amounts, aggregations of p62, and concentrated lysosomal biogenesis via promoting the TFEB transcript, as well as LAMP1 levels by reproducing diminished lysosomal action [59]. Augmented transcripts of Parkin and PINK1 create greater Parkin conscription to mitochondria, and a deficiency of the employment of the phagophore supported these clarifications. This late feature would propose an initiation of mitophagy and a barrier at the phase of mitophagosome construction. In the literature, investigators described aggregations of mitochondria discovered via superior TOMM20 levels and a raised quantity of mitDNA copies [59]. This previous machinery is represented in Figure 4.

Remarkably, the failure of autophagy development was similarly described in iPSC-derived cortical nerve cells gained from APOEε4 patients and APP (V717L) considered by decreasing amounts of LC3-II Bcl-1 and of autolysosomes and autophagosomes [43]. As evidenced by previous studies, other cells also exhibited decreased mitophagy, after which phosphorylations of ULK1 and TBK1 were decreased along with the amounts of many proteins associated with mitophagy paths [43]. Neuroblastoma cybrid cells (SH-SY5Y) shattered from their mitochondrial amounts. They are accompanied by mitochondria from patient thrombocytes with AD with a display of changed mitochondrial structure and action (improved ROSmit creations and decreased ATP) connected to weakened mitophagy, which itself is possibly associated with the reduced expression of PINK1 [60].

Remarkably, a genomic marker of compromised mitophagy was described in the peripheral fluids of some patients with AD syndrome, as demonstrated by a lessening of the autophagic ATG5 aspect and Parkin amounts in the circulating blood of AD patients [61], and a decline of Parkin content against a rise in the mRNA of LC3 and PINK1 at early stages in circulating blood [32]. These reports showed the advantages of using mitophagy transcriptomic performers as peripheral indices of AD. As indicated in a recent investigation, an ELISA method was established to recognize p-S65-Ub contents in diverse cellular conditions and described raised p-S65-Ub ranges in the frontal cortex of persons with AD compared to regular groups [62].

## 5. Mitophagy Failure Markers in AD Models

According to many previous genomic alterations in the brain of postmortem humans, it has been mentioned that the transcriptomic and proteomic screening (mRNA or proteins) of numerous important regulatory systems are recognized for contributing to mitophagy and/or autophagy manners (Table 1). Nevertheless, there are plentiful reports formed on individual associative explanations that connect the instruction of the transcripts of these indicators with syndrome phases. The research on human-derived cells has unraveled the possible consequence of molecular performers related to FAD and SAD forms (Table 1). However, the explicit repercussions of AD-related proteins, specifically Aβ, pTau and other APP-derived wreckages and genotypes of APOE in mitophagy mechanisms, were officially established in AD animal (in vivo) and cellular (in vitro) trials. Trials on cellular systems could support the display of autophagy/mitophagy fluidity, enumerating the crescendos of the authorization of precise mitochondrial indicators in the attendance or deficiency of an autophagy suppressor and/or utilizing mitophagy correspondents. As depicted in Table 1, we summarized the association of Aβ, Tau, APP-CTFs, and AD risk issues in mitophagy impairment in the mitochondria of a neuron based on the in vivo and in vitro trials.

### 5.1. Aβ Peptides

For a second time, researchers focused on the impact of Aβ peptides on mitophagy pathways, raising differing assumptions. The study of [71] has described that a 12 h exogenous use of Aβ_1–42_ peptides (7 µM) on a rat PC12 model produced a damaged mitophagy categorized via decreased amounts of LC3-II/I ratio, Bcl-1 Parkin, and PINK1, in addition to an aggregation of p62. Contrarily, [67] planned a longer (around one day) exogenous use of 5 µM Aβ_1–42_ peptide that produced a significant rise of LC3-II/I Parkin and Bcl-1 in PC12 cells. The same authors found that the upregulation of these genes, viz., LC3-II, Parkin, PINK1, and Bcl-1 transcripts, was connected to mitochondrial impairments, which was revealed by more significant ROS generation, decreased ΔΨmit, and lower ATP, and was detected in murine embryonic hippocampal neurons after treatment (3 days) [67]. Moreover, these data topics concern either a malfunctioning abolition of the impaired mitochondria by mitophagy connected with Aβ1–42, categorized via a faulty beginning of mitophagy, or a regular beginning of mitophagy monitored via a barrier at late phases of the manner. The oligomeric Aβ_1–42_ (oAβ_1–42_) administration of HEK293T (human non-neuronal cells) also instigated a stimulation monitored via a blockade of mitophagy, as verified by a rise of LC3-II/I proportion and Parkin and an aggregation of p62 in the mitochondrial division [68]. The final step of a malfunctioning mitophagy connected with oAβ_1–42_ use was supported by the statement of an aggregation of both mitochondria and autophagosomes [68]. The previous clarifications were authenticated according to in vivo trials. Certainly, the intracerebro ventricular injection of Aβ_1–42_ in rats generates decreased planes of Bcl-1, Parkin, and PINK1 and an aggregation of p62 [72]. Correspondingly, decreased mitophagy action was further stated in an Aβ_1–42_-neuron-transcprit *C. elegans* (CL2355) [53,60]. Remarkably, PINK1 upregulation can reduce Aβ_1–42_ and APP levels in in vivo models, thereby representing the capacity of PINK1 to release Aβ_1–42_-related imperfections [53]. In mice expressing hAPPswe/Ind, it was mentioned that soluble oAβ and Aβ peptides interrelate with AVs, triggering their weakened axonal retrograde carriage and their aggregation in neuron terminuses [73]. Guglielmotto and coworkers described that the moAβ (monomeric Aβ_1–42_) also prompts a barrier of the autophagy flux in distinguished SK-N-BE (human neuroblastoma cells). [46]. This feature was demonstrated by the aggregation of autophagosomes and p62 and potentiality decreased lysosomal response.

### 5.2. Tau Protein

Research on the potential effects of Tau protein on mitophagy has made certain conclusions that could seem contradictory. The neurotoxicity of NH_2_hTau (20–22 kDa, NH_2_-Tau fragment), present amid amino acids 26 and 230 of the lengthiest human tau isoform, destructively encourages mitochondrial impairment via mitophagy signaling [64]. The transcript of NH_2_hTau in advanced hippocampal primary nerve cells changes mitochondria assembly, prompts synaptic changes, and augments mitophagic fluidity, as proven via administration with suppressors in cooperation with autophagosome synthesis with lysosomal and lysosome protein declination [64]. Mitophagy’s genomic or pharmacological reticence also prohibited NH2hTau-intermediated damages [31,63]. Some of the above articles assumed that the aggregation of NH_2_hTau might contribute to synaptic damage in models of AD via aggravating mitophagy action. Nonetheless, other authors described the suppressor impact on the activation of mitophagyvia full-length human wild-kind Tau aggregation [51,74]. Moreover, the overexpression of hTau and the increase in pTau in the OMM portion increases ΔΨmit, consequently avoiding the maintenance of mitochondria PINK1 and the following Parkin enrollment [51]. As indicated in animal models, the upregulation of hTau repressed mitophagy in the neuroblastoma tissues of the *C. elegans* model [74]. As observed in studies, the mitophagy barricade existed not owing to a modification in the mitochondrial content of ΔΨmit but to an abnormal communication amid the prognosis motif of Parkin and Tau proteins inducing the weakening of its translocation to trigger the mitochondria impairment [74].

Recent research on *C. elegans* BR5270 strain showed an expression of the proaggregate F3ΔK280 hTau protein in nerve cells, similarly displaying mitochondria impairment and a decreased quantity of mitophagy actions [35]. Remarkably, to escape any imprecise assumption associated with upexpression approaches, a transgenic *C. elegans* model was reported to convey a single copy of hTau [65]. While hTau’s unique transcription appearance did not provoke noticeable or observable pathological indicators, a decrease in AD-associated PTM (post-translational alterations) of Tau displayed age-dependent neuronal [3,75] and behavioral morphological irregularities [76]. The same work described that the mutants of PTM required the capability to be involved in nerve mitophagy paths’ reconnection to mitochondria, which is stimulated via parquet components [65]. To review, these consequences confirmed that mitophagy damage might be connected to Tau pathological features. They strained that different assumptions could be haggardly conferring to the landscape of the Tau protein investigated and the methods and model applied to screen mitophagy.

### 5.3. APP-CTFs

The APP intracellular domain (AICD) produced via the cleavage of C99 or C83 wreckages by γ-secretase transcriptionally switches the manifestation of the main genes involved with AD syndrome [77]. Moreover, the AICD regulates mitophagy paths via the transcriptional stimulation of *PINK1* [44]. It depicted that the molecular paths involving mitophagy and PINK1 were represented via PS1, but not PS2, and that they activate the trans-stimulation of *PINK1* supporter via FOXO3a-dependent means, which increases PINK1 protein and mRNA via its connection to γ-secretase action and APP, but self-regulates the tensin and phosphatase homolog PTEN (a controller of PINK1 kinase action). Remarkably, various reports discovered a regulatory loop in which Parkin, which transcriptionally regulates PS2 and PS1 [78,79], represents an upstream of *PINK1* to stimulate the AICD-intermediated control of PINK1 transcript. Developing reports recommend that the primary aggregation of C99, rather than Aβ, is associated with mitophagy and autophagy faults in patients with AD. Therefore, C99 aggregations were indicated by to occur in numerous AD transgenic rat models and APP/KI mice protecting several or single APP/FAD mutations [79]. Significantly, current research testified that a C99 aggregation is associated with nerve cell susceptibility in human brains with AD syndrome. Many studies have established that C99 aggregations encourage the diminished lysosomal-autophagic actions, unconventionally, of Aβ [69]. This contrary influence seems to be owing to the mass of C99 within the massive relations singling among autophagic, endosomal, and lysosomal vesicle sheaths, possibly participating in behavioral shortfalls and synaptic impairment [76].

C99-intermediated endolysosomal impairment was shown in the iPSC of humans, resulting in embracing AD-related *PS1* or *APP* mutations demonstrating an endogenous APP transcript [76,80,81], and thus demonstrating that weakened autophagic-lysosomal tasks are not an object owing to a burden of mutated C99 or APP portions in upregulated models. In addition, some reports demarcated the dispensation of APP in MASs (mitochondria accompanying sheaths) and the aggregation of APP-derived rubbishes in this microdomain, affecting the roles of MASs [81,82]. The MAS was termed as a significant hotspot for mitophagy paths. Pending mitophagy stimulation, Bcl-1 and PINK1 re-establish in MASs to encourage phagosome construction [83]. Afterward, PINK1 phospho-ubiquitination and Parkin independent of the ER-mitochondria chain MFN2 detach mitochondria and license its autophagy and mitophagic degeneration [83]. Remarkably, there is a rising quantity of mitochondria and ER interaction locations as described in AD trials (cellular) and human (in vivo model) brains [84].

In a modern study [75], the authors described that APP-CTFs gather in the mitochondria-supported segment of neuroblastoma cells in human-articulating APP sheltering the Swedish familial mutation [75] that encourages APP-CTFs and Aβ construction. These authors also scrutinized mitochondrial assembly and its functionality modifications in these cellular systems. They described a stimulation of mitophagy’s first phases, categorized by improved levels of Parkin, PINK1, and the percentage of LC3-II/I. Nevertheless, other studies detected that the whole mitophagy manner was obstructed, as discovered by the absence of p62 degeneration, the amassing of some mitochondrial proteins (HSP10, HSP60, TOMM20, and TIMM23) and via weakened mitophagosome–lysosome synthesis [29,43,66]. Obviously, mitochondria assembly modification, ROS synthesis, and mitophagy disability observable traits were aggravated by the γ-secretase suppression. On the antagonistic, the suppression of β-secretase (that inhibits the synthesis of C99 and Aβ) inclines to improve mitochondria assembly and to liberate complex I action fault and ΔΨmit depolarization. Moreover, it was established that part of APP-CTFs on mitophagy miscarriage and the oversynthesis of ROSmit in cell lines (SH-SY5Y cells) articulate C99 part only. Similarly, APP-CTFs aggregated in the mitochondria of neurons before any recognition of amyloid signs in adeno-linked virus C99 (AAV-C99)-inoculated rats and in 3xTgAD mice [13,62,75].

Significantly, the γ-secretase suppression in presymptomatic mice (3xTgAD) aggravated mitochondrial assembly modifications and hindered the mitophagy route. These verdicts were freshly established in iNSC ancestral AD/PS1/C737A (in vitro model) [21]. Remarkably, the therapeutic shortcoming of the γ-secretase in AD-iNSC also degenerated the mitophagy and mitochondrial impairments, similarly to the impact of the PS1/PS2 double knockout-iNSC [21]. These findings confirmed the involvement of APP-CTFs, and Aβ individualistically, in mitophagy imperfections in patients with AD [75].

### 5.4. APOE4

APOE is a conserved glycoprotein mostly found in neurons, microglia, astrocytes, and cerebral tissues. Moreover, APOE is a fat carrier for cholesterol and its esters. Genetically, *APOE* is a metamorphic gene programming for three main APOE epsilon (APOEε) isoforms with variance attaching to fats. It is traditionally believed that APOEε2 concerns a diminished menace of AD together with the APOEε3 allele. Contrarily, a sole APOε4 allele raises the jeopardy of AD fourfold, which is linked to the mutual APOEε3/ε3 genotype, whereas the attendance of 2 APOEε4/ε4 alleles supplements the menace of AD by around 12 times. The influence of APOE4 on mitophagy paths was meticulously analyzed in past decades. Relative to APOE genotypes, it designated a greater aggregation of mitochondrial and accumulated proteins in T98G cells (glioblastoma) communicating APOE3 vs. APOE4 [85]. This manner accompanied a blockade of mitophagy and autophagy methods, probably owing to the necessity of APOE4 to synchronize lysosomal appearance and guideline DNA motifs, challenging TFEB and cooperating with the TFEB-intermitted transcriptional overexpression of *LAMP2*, *MAP1LC3B*, and *p62/SQSTM1* [85]. Relative transcriptomic scrutinization of the brain tissues of humans postmortem certainly exhibited that APOEε2/ε3 transporters, related to APOEε4/ε4 and APOEε3/ε4 carriers, presented the overregulation of *BNIP3*, *NBR1*, *OPTN*, *MAP1LC3B*, and *p62/SQSTM1* transcription [86]. Therefore, APOE4 mice exhibit greater mitochondria indication amounts (COX1 and TOMM40) and decreased cristae thickness in brain neurons compared to APOE3 mice [70]. These explanations propose a lessened mitophagic capability and a gathering of injured mitochondria associated with highly expressed APOE4. The examination of the fundamental transcriptomics machinery also designated a beginning of mitophagy reproduced by decreasing sliced PINK1, a symbol of decreased ΔΨmit, and via preeminent Parkin contents in mice with APOE4 mutants [70].

Mitophagy seems to be obstructed in late phases, as demonstrated by augmented mitochondria indicators and p62 [70]. Additionally, APOE4-articulating astrocytes displayed weakened mitochondrial function. One study displayed decreased mitophagy and separation categorized by decreased LC3-II proteins and active Parkin, added p62, and ridding ubiquitination and proteasomal/lysosomal degeneration of self-motivated mitochondrial proteins [87]. Lastly, iPSC-derived cortical nerve cells, taken from APOEε4/ε4 of patients with AD, decreased the phosphorylation of mitophagy architects ULK1 and TBK1 and decreased transcripts of Bcl-1, PINK1, and LC3-II, relative to regular nerve cells [59]. These explanations may propose a barricade of mitophagy at the original periods of its initiation. In addition, it must be eminent that the APOEε4 SAD jeopardy feature is also related to augmented cholesterol amounts in the serum and the overflow of cholesterol to nerve cells and atherosclerosis [88]. Extraordinarily, it was updated and determined that augmented intracellular cholesterol levels could influence mitophagy paths [89]. Certainly, severe upgrades of cholesterol in neuroblastoma SH-SY5Y cells damage the mitophagy flux by obstructing mitochondria distribution to lysosomes, which are smooth in the occurrence of oAβ_1–42_ or the mitochondrial uncoupler CCCP [89].

### 5.5. Ca^2+^ Ions in the Regulation of Mitophagy

2Ca^2+^ is a noteworthy second delegate that can inaugurate the cellular life and death paths in mitochondria. The assembly between AD and calcium was detected over the last few years [90]. Still, updated hypotheses have been evidenced and strongly involve a function for calcium in the pathophysiology of AD [91]. Furthermore, mitochondrial Ca^2+^ modifications may affect the functionality of other brain neurons contributing to memory development and association [92]. AD is characterized by mutations in *PSEN1/2,* which lead to the augmentation of the opening of mitochondrial permeability transition pore (mPTP) Ca^2+^-releasing channels, producing an extreme statement of Ca^2+^ from this store [92,93]. The accumulation of Ca^2+^ in the mitochondria usually leads to cell death compelled via necrosis and apoptosis [94]. Moreover, mitochondrial Ca^2+^ excess can impair mitochondria via mitophagy.

## 6. Genetic Attributes Targeting Mitophagy

Plentiful hereditary attributes targeting constituents of the mitophagy mechanism were applied to relieve toxic abuses associated with the aggregation of disease-associated impaired mitochondria. As illustrated by [52], the upregulation of Parkin via applying lentivectors in fibroblasts with SAD increases autophagy fluctuation and, importantly, renders the degeneration of aggregating mitochondria. The upregulation of Parkin also amends mitochondrial jobs via the retrieval of ΔΨmit [52]. Previous investigations have examined the effects of PINK1 in a preclinical AD exemplary presentation and showed that intraneurons in brain tissue cause stereotaxic vaccinations of AAV2-hPINK1 in transgenic rats at six months, exhibiting an upregulation of hAPP comportment Indiana (V717F), and that Swedish mutations trigger the stimulation of mitophagy via an over-regulation of both NDP52 and OPTN mitophagy receptors [53]. In another statement, [68] indicated that the overexpression of Parkin in Aβ-administrated HEK293T cells could escalate translocated and cytosolic Parkin contents and LC3-II content, and decrease p62 contents in mitochondria in patients around the world, representing a release of mitophagy as mentioned in the previous cells. The upregulation of Parkin also weakened Aβ-instigated mitochondrial division and impairments as demonstrated by the dropping of ROS generations and the promotion of ΔΨmit; complex enzymes, including I, II, and IV; and ATP synthesis. Significantly, mice injected with PINK1 have decreased Aβ pathology (Aβ gathering and the number of plaques) in the brain tissues, enhanced learning memory and synaptic function, a regular mitochondrial role, and decreased synaptic damage [53]. The DISC1 protein is recognized to control retrograde and anterograde axonal mitochondria conveyances. Lately, however, it has been revealed as a potential part of a mitophagy receptor via an LIR-motif-dependent necessity of LC3 [71]. In vitro, CCCP-instigated mitophagy or DISC1 siRNA inverted oAβ_1–42_, whereas DISC1 upregulated and activated an LC3-dependent mitophagy and salvaged oAβ_1–42_-instigated synaptic and mitochondrial imperfections [71].

In boosting these previous verdicts, as indicated in vitro, the overexpression of DISC1 in APP/PS1 (APPswe/PS1ΔE9) mice decreased synaptic loss, amyloid sign density, and cognitive shortcomings via the elevation of mitophagy [71]. In brain tissues, the connector protein Snapin contributes with the petrol-powered protein Dynein reversing the detached mitophagosome carriage to the other segment, such as the soma, for completing mitophagic degeneration [73]. The absence of Snapin in murine neuron tissues reiterates AD synaptic imperfections by eliminating Snapin-intermediated reversing transportation and instigating presynaptic mitophagic stress [35,72,73]. On the antagonistic, the overexpression of Snapin weakened the stress of synapses reversal and presynaptic mitochondria in a hAPPswe/ind AD rat through expediting the reversing transportation of axonal mitophagosomes [81]. To conclude, the overexpression of Miro1 messenger released injured mitochondrial motility and morphology and diminished oAβ-intermediated mitophagy paths [35,72].

## 7. Genetic and Epigenetic Modifications in AD

The genome contains the two categories of genetic evidence and epigenetic evidence provided by DNA sequences. The epigenetic exhibits the relationship of epigenetic alterations with the regulation of gene expression and differentiation, as well as genomic modifications in gene activity, without changing the DNA sequence [95,96]. The genome of mitochondria shortens the safeguard of histones and multifarious DNA repair machinery [97,98] due to its vulnerable to OS, resulting in fluctuations in mtDNA mutation. Mitochondrial epigenetic alterations comprise noncoding RNA, DNA methylation, and post-translational modifications of nucleoid-related proteins [99,100]. Experiments have revealed that the methylation of the *D-loop* region, *12S rRNA*, *MT-ND1*, *COX II*, and *CYTB* were hypermethylated. At the same time, the mtDNA copy number was decreased in the hippocampal tissue of AD in animal models [95]. Moreover, controlling global hydroxymethylation (5-hmC) and DNA methylation (5-mC) could increase the transcriptomics of some histone deacetylase enzymes (*Hdac1* and *Hdac2*), which are associated in the cognitive impairment of AD in mice models [101]. Using the recently established m1A-quant-seq process, Shafik et al. mapped brain-rich N1-methyladenosine (m1A) RNA alterations in the brain cortex (5XFAD) of a mouse model of AD. They found that the levels of m1A in mitochondrial and cytoplasmic tRNAs are lost in AD, leading to a more destructive AD phenotype [102].

Additionally, mt-tRNA methylation could affect the transcript of nuclear genes via deteriorating signaling; however, the exact machinery regarding this topic is unclear [97]. Collectively, various mitochondrial epigenetic types of machinery have central roles in the incidence and progress of AD and the interruption of the dynamic balance of mtDNA methylation and demethylation, which are the most explored mitochondrial epigenetic modification. Still, investigators have not yet figured out whether the mtDNA methylation modifications are the result of AD or the reason that AD occurs. Additional investigation is required to clarify this.

## 8. Therapeutic Approaches

Globally, AD is the utmost prevailing reason for dementia and a substantial problem to the entire healthcare structure. For several decades, scientists focused on research regarding the machinery of AD and its causes. Furthermore, there are presently no identified medications for treating or preventing AD; however, some molecules could help in reducing the severity of AD. This section will explore the potentiality of using some molecules as therapeutic approaches toward AD. Based on the literature collected for this review, mitophagy enhancers are hopeful therapeutic medications to remedy mitophagy paths [66] in patients with AD. In Table 2, we summarized the findings of the main therapeutic approaches for treating AD via the mitophagy process described in the present review.

### 8.1. Actinonin (AC)

AC is a natural molecule with different biological actions, viz., antibacterial and antitumor activities [43]. In the case of using AC treatment in an AD nematode model, the mode of action might be ascribed to its ability to prevent memory faults and decrease Aβ burden and APP-CTF load. In APP/PS1 mice, AC reestablished mitochondria functions and morphology and greater synapse quantity by inspiring mitophagy [43]. Remarkably, AC stimulated mitophagy in microglia by decreasing neuroinflammation and motivating Aβ inscription allowance. In this way, AC displayed moderate defensive impacts alongside mutant Aβ- and APP-instigated mitochondrial impairment and synaptic toxicity in AD syndrome [29].

### 8.2. Urolithin A (UA)

UA is widely derived from ellagitannins phytochemicals and is recognized to have valuable impacts on mitochondrial functions and homeostasis via the inspiration of mitophagy in animals and mammalian species [112]. Currently, the influence of UA has been shown in different animal models (in vivo) with AD replicas and has clarified important enhancements of pathological variables via an introduction of mitophagy [43]. Henceforth, the administration of *C. elegans* articulation of APP/PS1 and Aβ_1–42_ mice with UA decreased Aβ cognitive failure and pathology by instigating Parkin/PINK1-dependent mitophagy. Additionally, UA was revealed to save mitochondria functional and structural faults and escalate synapse amounts. Furthermore, UA reduced mitochondrial impairment in microglia, inverted inflammatory actions, and inspired the phagocytic consent of Aβ signs. UA administration also improved memory and significantly decreased Tau hyperphosphorylation in mice (3xTgAD) [33]. The first human clinical trial [103] used UA (0.5–1 g for one month) in healthy deskbound aging persons and confirmed its protection and advantage by controlling mitochondrial genomic alterations.

### 8.3. Resveratrol (3,5,4′-Trihydroxy-Trans-Stilbene; RES)

RES is a natural phenolic molecule performing as a robust ROS hunter, an iron chelator, and a mitophagy and autophagy stimulator [104]. RES shows many positive impacts for AD. RES defends PC12 cells from OS, apoptosis, death, and mitochondrial impairment caused by Aβ_1–42_ by stimulating mitophagy paths [15]. The prolonged oral management of phenolic compounds in APP/PS1 mice enhanced mitochondrial functions and brain memory, triggered AMPK and SIRT1 paths, and decreased Aβ content [104]. Nevertheless, this fragment has a main clinical disadvantage since it is metabolically unsteady and, consequently, proposes a depressed bioavailability in water [113]. The authors suggested that using the nanoform of RES can enhance the bioavailability and improve the efficacy of treating AD. Other molecules have been widely used as mitophagy enhancers. RES (stilbenoid) and Kaempferol (flavonoid) are two natural molecules lately recognized as mitophagy stimulators by a machine learning awning, and authorized in vivo and in vitro [114]. Both of these molecules decreased the contents of APP-CTFs, pTau, and Aβ in cells, larva, or mice models of AD. RES and Kaempferol reinstated memory shortages in Aβ and Tau of AD trial replicas [114]. These advantageous impacts were due to mitophagy’s encouragement via an overexpression of some mitophagy performers and the elevation of the mitochondrial division [15,114]. As reported by [104], Kaempferol has previously been identified as capable of forming junctions in brain barriers and may decrease the jeopardy of emergent AD.

### 8.4. NAD^+^ Boosters

NAD^+^ is a cofactor for many molecules, including those in the Sirtuins family (SIRT1, 3, 6, and 7), which is capable of encouraging wide-ranging mitophagy and autophagy via various paths [105]. Both NMN (nicotinamide mononucleotide) and NR (nicotinamide riboside) are vigorous stimulators of mitophagy paths [115]. In the animal model (*C. elegans* expressing Aβ_1–42_), NMN had greater mitophagy paths, including PINK1/Parkin-dependent, and restored memory faults [43]. At the same time, NR caused mitophagy, decreased proteotoxic stress and Aβ burden, and enhanced lifespan and health [106]. Moreover, NR improved OXPHOS, LC3, and PINK1 mRNA levels, decreased cortical Aβ deposits, and enhanced cognitive utilities in mice with APP/PS1 [106]. Numerous clinical experiments continue to evaluate NR’s impact on brain health, OS, cognition, or CSF pTau contents in MCD (mild cognitive damage) and patients with AD. Approaches, such as NAD^+^ level restoration in neuronal tissues via the amplification of NAD^+^ precursors, including NR and NMN, could stimulate mitophagy inspiration and hold possible clinical results as medications against AD.

### 8.5. Plant-Derived Extracts

Tetrahydroxy is a glycoside isolated from the Polygonum multiflorum (which has been used in Chinese medicine) and was termed to have neurodefensive properties in AD [107]. This favorable impact appears to happen by targeting mitophagy and autophagy via the AMPK/PINK1/Parkin paths [59]. Moreover, the central stimulator of Acorus tatarinowii (Schott herb) is β-Asarone, which activates mitophagy and autophagy and relieves Aβ_1–42_ cytotoxicity in vitro [71]. Reports from in vivo studies described that β-Asarone had valuable properties alongside Aβ-linked pathology via the stimulation of autophagy in APP/PS1 mice [116] and reduced Aβ_1–42_ in neurons. Additional work, rather, suggested that β-Asarone enhanced knowledge and memory in mice inoculated with Aβ_1–42_ by endorsing mitophagy [72]. In addition, Trehalose is a natural biose performing as a TFEB and lysosomal stimulator, an mTOR-independent autophagy inducer. It guards mitochondria against OS via a BNIP3-instigated mitophagy [117]. In addition, trehalose management in vivo presented neuroprotective assistances in various AD mice examples [118]

### 8.6. Spermidine (SP)

SP is a spiny natural organic compound recognized to lengthen the lifecycle of nematodes flies, yeast, and rats via stimulating autophagy [113]. In human fibroblasts, it also motivates the Parkin/PINK1 mitophagy paths [119]. Interestingly, it has lately been renowned for stretching lifecycles and prohibiting memory damage in a PINK1/Park signaling-dependent approach in an animal model, such as *C. elegans* evidencing human Tau and Aβ [108,109]. In humans, medical trials in aging matters with MCI determined that SP may increase neuron-dependent recollection [108].

### 8.7. Circadian Hormone

Melatonin regularly clutches numerous physiological roles, among which is neuroprotection ability. Only one study has reported the potential of melatonin as a mitophagy enhancer. In [110], prolonged oral management of melatonin in PS1 mice evidenced a neuroprotective ability by enhancing mitochondrial assembly and functionality and vindicating the extreme mitophagy via a decrease in the transcripts of mitophagy indices (LC3-II/LC3-I, Parkin, PINK1) and of the amount of autophagic vesicles. Moreover, melatonin administration similarly down-expressed APP, treating memory faults and enhancing spatial learning [110].

### 8.8. Lifestyle Strategies

PE (Physical exercise) was previously applied in the environment of some mental or metabolic syndromes and has been stated to be favorable in AD syndrome [6]. Still, the specific machinery remained unidentified. In AD mouse models, PE exhibited helpful properties on synaptic plasticity modifications, reestablished neuroprotective feature altitudes, amended cognitive shortages, and decreased Aβ assembly [120]. Frequent trials recommended that PE be represented via the SIRT1/PINK1/Parkin cascade way [121,122]. In addition, PE for APP/PS1 mice augmented mitophagy, improved knowledge and memory capabilities, synaptic action, Aβ burden, and mitochondrial functionality, and reduced OS markers [123]. In addition to PE, CR (caloric restriction) is a robust stimulator for the mitophagy process [124]. In AD mice models, CR upregulated NAD^+^ and SIRT1 contents, decreased hippocampal Tau and Aβ capacity and alleviated behavioral scarcities [119]. A screening of the literature shows that there is scarce information on this topic and further trials are required.

### 8.9. Nanotherapeutic Approaches

For many decades, and as shown in the previous sections, many natural molecules have been widely used in treating AD. However, these molecules have exhibited few soluble, bioavailable, and sustainable applications. To overcome these problems, scientists used nanocarriers to improve prolonged circulation, drug bioavailability, and the overpowering/bypassing of biological walls. Plentiful reports used different nanoforms, such as resveratrol-loaded nanoliposomes (RLNB) [111] and luteolin nano-bilosomes (LNB) (50 mg/kg) [28] for treating AD in rats. The authors of [28,111] clarified that mice administered with the RLNB and LNB displayed a significant reduction in IL-6, COX2, Tau, and Aβ contents compared to the drug suspension, including luteolin or RES [28]. Moreover, [125] observed a noticeable cognitive improvement, the improvement of memory, and a substantial decrease in β-Tau and Aβ in mice administrated with artichoke extract, and a superior scope with chitosan-coated artichoke-loaded nanoform. In conclusion, these observations act as a signpost showing that natural molecules coated with bilosomes could be considered new delivery schemes that present great possible applications to boost bioavailability and stability.

## 9. Conclusions and Future Remakes

The present study summarizes recent investigations involving the effect of mitophagy miscarriage on AD pathogenesis. Documents on the brain tissues of human postmortem and derived cells emphasized that mitophagy miscarriage is communal to both FAD and SAD events. Investigations in preclinical AD models believably confirmed that numerous AD transistors (APP-CTFs, Tau, Aβ, PS1, and APOE4 mutants) impair mitochondria, and that some genetic and epigenetic modifications lead to the degeneration of mitochondria assembly and functionality. Because of the genomic aspect, the genetic targeting of scarce mitochondrial mitophagy disintegration by upregulating proteins, which is involved at various stages of this method, caused a substantial release of numerous AD mitophagy. This verification boosted the knowledge that releasing mitophagy in patients with AD could be a productive therapeutic approach. Based on many results, plentiful molecules can be used to encourage mitophagy, which has revealed constructive impacts in preclinical AD representations. These molecules have also been managed in AD or healthy patients in many phases in clinical experiments (NMN, NR, UA, melatonin, and spermidine). Recently, nanocarriers were applied for AD remedy; however, there is scarce data regarding this topic, so further explorations are required to enhance the efficacy of drug delivery. Nevertheless, enhancing these drug applicants, their bioavailability (i.e., biodegradation and the passaging of the BBB), and their therapeutics and interface with their goals at the proper place, is necessary. Recently, advancements in machine learning were used to improve traditional time-up-taking attitudes for drug discovery. In the mitochondrial singling assumption. Mitochondrial impairments are the main actions in AD syndrome participating in downregulating pathological molecular corridors, fast-tracking syndrome development. Hence, is it plausible to encourage mitophagy in healthy or at-risk individuals to reduce and avoid AD progress? Will a sole mitophagy-motivating molecule be sufficient or must we envisage the management of mitophagy-motivating medications together with CR or PE lifestyle fluctuations, or even in combination with eminent in-test approaches (i.e., pyroglutamate Aβ, monoclonal antibodies alongside Aβ_1-42_, or pTau classes)?

## Figures and Tables

**Table 1:** Association of Aβ, Tau, APP-CTFs, and AD risk issues in mitophagy impairment in vivo and in vitro.

Item	Models	Mode of Action	Findings	References
hTau	HEK293T cells (in vitro)	↑ COX IV proteins and TOMM20	↑ Mitochondrial mass	[51]
	Tg2576 mice (in vivo)	Increased Parkin protein	↑ Parkin recruitment to mitochondria	[63]
NH2hTau	Rats (hippocampal neurons)	↓ mitDNA (mtND2)/gDNA (Htert) ratio	↑ Mitochondrial degradation	[64]
		↓ CytC, ATPB, TOMM20, VDAC1, Mn-SODII, and TIMM23 proteins	↑ Lysosomal degradation	
		↑ LC3-II protein	↑ Phagophores number	
		↑ Parkin protein in mitochondria and ↑ PINK1 protein	↑ PINK1/Parkin activation	
		↓ p62 protein	↑ Lysosomal degradation	
hTau F3ΔK280	*C. elegans*	↓ Mitolysosomes number	↓ Mitolysosomes formation	[65]
	*C. elegans*	↓ LC3-BNIP3/NIX colocalization	↓ Phagophores recruitment	[43,66]
Aβ1-42	PC12 cells	↑ Bcl-1 and ↑ Parkin mRNA and proteins	↑ Mitophagy initiation	[31,67,68]
		↑ LC3 protein and LC3-II/I ratio	↑ Phagophores number	
		↓ Bcl-1 protein	↓ Mitophagy initiation	
		↓ PINK1 and Parkin proteins	↓ PINK1/Parkin activation	
		↓ LC3-II protein	↓ Phagophores number	
		↑ p62 protein	↓ Lysosomal degradation	
mAβ1-42	SK-N-BE cells (1 µM)	↑ Bcl-1 protein	↓ Mitophagy initiation	[46]
		↑ LC3-II protein	↑ Phagophores number	
		↑ Autophagosomes number ↑ p62 protein ↓ CTSD activity	Autophagosomes accumulation ↓ Lysosomal degradation	
oAβ1-42	HEK293T cells	↓ Mitolysosomes number	↓ Mitophagy	[68]
		↑ mitochondria number	Mitochondria accumulation	
		↑ Parkin protein	↑ Parkin activation	
		↑ LC3-II protein	↑ Phagophores recruitment	
		↑ p62 protein	↓ Lysosomal degradation	
APP-CTFs				
AICD	HEK293T	↑ PINK1 mRNA and protein	↑ PINK1/Parkin activation	[44]
		↑ LC3-II protein	↑ Phagophores number	
C99 and C83	SH-SY5Y APPswe ± γ-sec inhibitor	↑ LC3-II protein	↑ Phagophores number	[69]
		↓ Mature CTSB protein ↓ CTSB activity	↓ Lysosomal degradation	
	Astrocytes from hAPOEε4/ε4 mice	↔ p62 protein ↓ Mitochondria cristae density	↓ Lysosomal degradation	[70]
		↓ PINK152 protein ↑ Parkin protein	↑ PINK1/Parkin activation	
		↑ TOMM20 and TOMM40 proteins	↑ Mitochondria accumulation	

↑, increased; ↓, decreased; ↔, stable.

**Table 2:** The main therapeutic approaches for treating AD via the mitophagy process described in the present review.

Compound	Model	Mode of Action	References
Actinonin (AC)	AD nematode model and mice	- Prevents memory faults. - Decreases Aβ burden and APP-CTFs load. - Prevents cognitive damage in APP/PS1 mice via microglial phagocytosis of extracellular Aβ plaques. - Inhibition of neuroinflammation.	[43]
	Immortalized mouse primary hippocampal (HT22)	- After treatment with AC as a mitophagy enhancer, cell survival was significantly increased in HT22 cells. - Mitochondrial fusion length was increased, while the mitochondria number and fragmentation were decreased in HT22 cells. - Mitophagy genes were increased in HT22 cells.	[29]
Urolithin A (UA)	AD nematode model and mice	- UA decreased Aβ cognitive failure and pathology by instigating Parkin/PINK1-dependent mitophagy. - UA reduced mitochondrial impairment in microglia, inverted inflammatory actions, and inspired the phagocytic consent of Aβ signs. - UA administration improved memory and decreased Tau hyperphosphorylation in the mice (3xTgAD) significantly.	[33,43]
	Human clinical trial	- UA controlled plasma acylcarnitines and mitochondrial genes in the skeletal muscle of aging persons. - UA triggered a molecular signature of enhanced cellular and mitochondrial health after regular oral intake in aging persons.	[103]
Resveratrol (RES)	Mouse model	- RES defends PC12 cells from OS, apoptosis, death, and mitochondrial impairment caused by Aβ_1–42_ by stimulating mitophagy paths. - Decreased the contents of APP-CTFs, pTau, and Aβ in cells, larva, or mice models of AD. - Reinstated memory shortages in both Aβ and Tau of AD trial replicas.	[15,104]
NAD^+^ boosters	Animal model (Aβ_1–42_) *C. elegans*	- Nicotinamide mononucleotide has greater mitophagy paths, including PINK1/Parkin-dependent and restores memory faults. - Nicotinamide riboside caused mitophagy, decreased proteotoxic stress and Aβ burden, and enhanced lifespan and health.	[105,106]
	Mice with APP/PS1	- Improved OXPHOS, LC3, and PINK1 mRNA levels, decreased cortical Aβ deposits, and enhanced cognitive utilities in mice with APP/PS1.	[106]
Plant-derived extracts	In vitro cell line	- *Polygonum multiflorum* has shown neurodefensive properties in AD by targeting mitophagy and autophagy via the AMPK/PINK1/Parkin paths. - *Acorus tatarinowii* (Schott herb) was stimulated by β-Asarone, activated mitophagy and autophagy, and relieved Aβ_1-42_ cytotoxicity in vitro. - β-Asarone enhanced knowledge and memory in mice inoculated with Aβ_1–42_ by endorsing mitophagy.	[59,72,107]
Spermidine	*C. elegans*	- Spermidine stretched the lifecycle and prohibited memory damage in a PINK1/Park signaling-dependent approach in an animal model such as *C. elegans* evidencing human Tau and Aβ	[108,109]
Circadian hormone	PS1 mice	- Melatonin has a neuroprotective ability via enhancing mitochondrial assembly and functionality and vindicating extreme mitophagy via a decrease in the transcripts of mitophagy indices (LC3-II/LC3-I, Parkin, PINK1) and in the amount of mitophagic vesicles.	[110]
Nanotherapeutic approaches	Mice model	- Mice administered with the luteolin and resveratrol nanoliposomes displayed a significant reduction in IL-6, COX2, Tau, and Aβ contents when compared with the drug suspension, including luteolin or resveratrol.	[28,111]

## Data Availability

All data presented in this review are collected from other previous studies.

## Abbreviations

TauP (Tau protein); Aβ (beta-amyloid); APOE (Apolipoprotein gene); PSEN 1 and 2 (presenilin 1 and 2); OM (outer membrane); ATP (adenosine triphosphate); OXPHOS (oxidative phosphorylation); IM (inner membrane); ROS (reactive oxygen species); OS (oxidative markers); PP2A (phosphatase 2A); GSK3β (glycogen synthase kinase); CCO (cytochrome c oxidase); ETC (electron transport chain); LPO (lipid peroxidation); MDA (malondialdehyde); 8-OHdG (8-hydroxydeoxyguanosine); 8-OHdG (8-hydroxy-2′-deoxyguanosine); HNE (4-hydroxy-2-trans-nonenal); 3-NT (3-nitrotyrosine); PC (protein carbonyls); GLUT4 (transporter type 4); mitDNA (mitochondria DNA copy number); AVs (autophagic vacuoles); optineurin (OPTN); AMPK (AMP-activated protein kinase); PINK1 (PTEN-induced kinase 1); AICD (APP Intracellular Domain); MASs (mitochondria accompanying sheaths); HSP10 (heat shock protein 10); AAV-C99 (adeno-linked virus C99); APOEε (APOE epsilon); LAMP2 (lysosomal-associated membrane protein 2); *CSNK1A1* (casein kinase 1, α 1); SQSTM1/p62 (sequestosome 1); *MAP1LC3B* (Microtubule Associated Protein 1 Light Chain 3 Beta); AC (Actinonin); UA (Urolithin A); RES (Resveratrol); NMN (nicotinamide mononucleotide); NR (nicotinamide riboside); PE (Physical exercise); resveratrol-loaded nanobilosomes (RLNB); luteolin nano-bilosomes (LNB).
